# A new sodium dimangnesium trivanadate, NaMg_2_V_3_O_10_

**DOI:** 10.1107/S160053680800322X

**Published:** 2008-02-20

**Authors:** Brahim Ayed, Amor Haddad

**Affiliations:** a Institut Préparatoire aux Études d’Ingénieur de Monastir, Avenue Ibn-El-Jazzar, 5019 Monastir, Tunisia; b Départament de Chimie, Faculté des Sciences de Monastir, 5000 Monastir, Tunisia

## Abstract

A single crystal of NaMg_2_V_3_O_10_ has been prepared by solid-state reaction at 1173 K. The [Mg_2_(V_3_O_10_)]^−^ anions are built up from edge-sharing MgO_6_ octa­hedra to form [Mg_4_O_18_] units, which are linked to each other by trivanadate groups (V_3_O_10_). The Na^+^ ions are located in the tunnel space.

## Related literature

For related literature, see: Barbier (1988 ▶); Brown & Altermatt (1985 ▶); Gopal & Calvo (1974 ▶); Krishnamachari & Calvo (1971 ▶); Mitiaev *et al.* (2004 ▶); Murashova *et al.* (1988*a* ▶, 1988*b* ▶); Ng & Calvo (1972 ▶); Saux & Galy (1973 ▶).

## Experimental

### 

#### Crystal data

NaMg_2_V_3_O_10_
*M* *_r_* = 384.42
Triclinic, 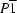

*a* = 6.7369 (1) Å
*b* = 6.7553 (1) Å
*c* = 9.6222 (1) Å
α = 104.325 (1)°
β = 100.604 (1)°
γ = 101.696 (1)°
*V* = 402.63 (1) Å^3^
*Z* = 2
Mo *K*α radiation
μ = 3.66 mm^−1^
*T* = 293 (2) K
0.4 × 0.07 × 0.03 mm

#### Data collection

Enraf–Nonius CAD-4 diffractometer
Absorption correction: ψ scan (North *et al.*, 1968 ▶) *T*_min_ = 0.871, *T*_max_ = 0.965 (expected range = 0.809–0.896)
3118 measured reflections
1712 independent reflections
1415 reflections with *I* > 2σ(*I*)
*R*_int_ = 0.049
2 standard reflections frequency: 120 min intensity decay: 0.4%

#### Refinement

*R*[*F* ^2^ > 2σ(*F* ^2^)] = 0.033
*wR*(*F* ^2^) = 0.095
*S* = 1.06
1712 reflections
146 parameters
Δρ_max_ = 0.73 e Å^−3^
Δρ_min_ = −1.18 e Å^−3^

### 

Data collection: *CAD-4 EXPRESS* (Duisenberg, 1992 ▶; Macíček & Yordanov, 1992 ▶); cell refinement: *CAD-4 EXPRESS*; data reduction: *MolEN* (Fair, 1990 ▶); program(s) used to solve structure: *SHELXS97* (Sheldrick, 2008 ▶); program(s) used to refine structure: *SHELXL97* (Sheldrick, 2008 ▶); molecular graphics: *DIAMOND* (Brandenburg, 1998 ▶); software used to prepare material for publication: *SHELXL97*.

## Supplementary Material

Crystal structure: contains datablocks I, global. DOI: 10.1107/S160053680800322X/br2067sup1.cif

Structure factors: contains datablocks I. DOI: 10.1107/S160053680800322X/br2067Isup2.hkl

Additional supplementary materials:  crystallographic information; 3D view; checkCIF report

## Figures and Tables

**Table 1:** Selected bond lengths (Å)

V1—O4	1.672 (3)
V1—O6	1.688 (3)
V1—O2	1.704 (3)
V1—O1	1.806 (3)
V2—O9	1.643 (3)
V2—O10	1.694 (3)
V2—O7	1.717 (3)
V2—O5	1.848 (3)
V3—O8	1.642 (3)
V3—O3	1.655 (3)
V3—O1^i^	1.757 (3)
V3—O5^ii^	1.827 (3)

Symmetry codes: (i) 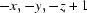; (ii) 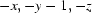.

**Fig. 1.**
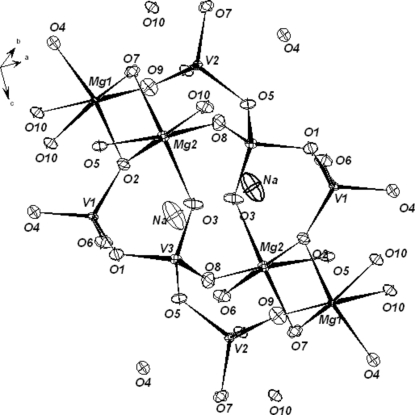
A projection of NaMg2V3O10, showing the displacement ellipsoids.

**Fig. 2.**
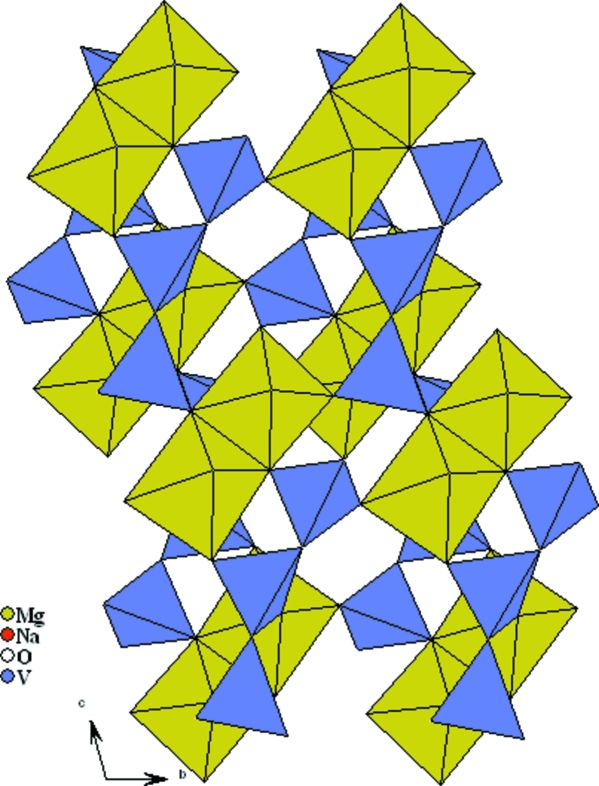
View showing the ribbons running along the [011] direction.

**Fig. 3.**
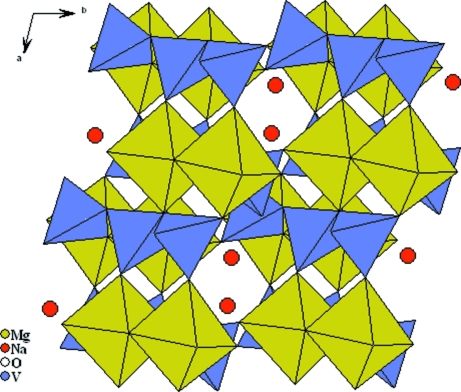
Projection of the structure of NaMg2V3O10 along [001] direction.

## supplementary crystallographic information

### Comment

The synthesis and structural characterization of new materials characterized by mixed open frameworks of MO_6_ octahedra and XO_4_ tetrahedra sharing edges and/or corners delimiting tunnels where cations are located and study of their properties are an active area of research in solid state chemistry, due to their interest in the fields of catalysis, ion exchange and ion conduction. In the system MgO–V_2_O_5_, a small number of compounds have been structurally characterized now, namely, Mg_2_V_2_O_7_ (Gopal & Calvo, 1974), MgV_2_O_6_ (Ng & Calvo, 1972), Mg_3_(VO_4_)_2_ (Krishnamachari & Calvo, 1971) and MgV_3_O_8_ (Saux & Galy, 1973). The same is true for the inclusion of alkali elements into this system, to our knowledge, only the structure of NaMg_4_(VO_4_)_3_ (Murashova *et al.*, 1988*a*), LiMg(VO_4_) (Barbier, 1988), K_2_MgV_2_O_7_ (Murashova *et al.*, 1988*b*) and Na_6_Mg_2_(V_4_O_15_)(Mitiaev *et al.*, 2004) have been determined. Extending our investigation a new magnesium trivanadate, NaMg_2_V_3_O_10_ has been prepared by a conventional solid-state reaction and characterized by single-crystal X-ray diffraction. The structure of NaMg_2_V_3_O_10_ consists of MgO_6_ octahedra and VO_4_ tetrahedral sharing corners and edges to form a three-dimensional framework. The Na^+^ are located in the tunnels space. A projection of the structure, showing the displacement ellipsoids, is presented in Fig 1. The Mg_2_ (V_3_O_10_)]^-^ anions are built up from edge sharing MgO_6_ octahedra to form [Mg_4_O_18_] units, which are linked to each other by trivanadate groups (V_3_O_10_). The Mg_4_O_18_ basal unit is built up by the edge linkages of four MgO_6_ octahedra, Such a unit is formed by two kind of divalent-metal cations, one labeled as Mg1 share edge with another labelled Mg2 forming Mg_2_O_10_ dimers, repetition of the dimers ensured by centres of symmetry on the shared edge between two Mg1O_6_ leads to the formation of Mg_4_O_10_ unit. Each trivanadate is formed by three tetrahedra, V1O_4_, V2O_4_ and V3O_4_, interconnected through the corners O1 and O5. The V1, V2 and V3 tetrahedron shares the eight remaining O-atom corners with five Mg_4_O_18_ units forming ribbons running along the [011] direction (Fig. 2). The projection of the structure along the [001] show that the trivanadate groups and the MgO_6_ polyhedra form six side tunnels running along [001] in which Na^+^ cations are located (Fig3). The geometry of the VO_4_ tetrahedra is close to that generally observed. Two groups of distances can be distinguished. The V—O bonds corresponding to the two V—O—V bridges of the V_3_O_10_ groups are the largest one. Consequently, the two external tetrahedra V1 and V2 present one long V—O distance and three smaller ones. Whereas the cental V3 tetrahedron has two long and two shorter. The Mg atoms are surrounded by six O atoms and the sodium cations exhibit a sixfold coordination. The bond valence sums determined using the Brown & Altermatt (1985) formulation are in the agreement with the formal charges deduced from the chemical formula: 5.088, 5.020, 5.100 from V1 to V3 respectively; 0.858 for Na; 2,188, 1.960 for Mg1 and Mg2 respectively, and ranging from 1.928 to 2.197 for the oxygen atoms.

### Experimental

The starting materials for synthesizing NaMg_2_V_3_O_10_ were NaVO_3_, V_2_O_5_ and Mg(NO_3_)_2_.7H2O. The stoichiometric mixture was heated in air, in a platinum crucible, after a progressive heating to 873 K, the mixture was heated at 1173 K, cooled to 773 K at rate of 5 K.hr-1 and then to room temperature. The parallelepiped crystals obtained after washing with hot water were brown. Quantitative analysis of these crystals, by electron microscope probe, revealed that they contain sodium, magnesium and vanadium.

### Crystal data

**Table:**

NaMg_2_V_3_O_10_	*Z* = 2
*M**_r_* = 384.42	*F*_000_ = 368
Triclinic, *P*1	*D*_x_ = 3.171 Mg m^−^^3^
Hall symbol: -P 1	Mo *K*α radiation λ = 0.71073 Å
*a* = 6.7369 (1) Å	Cell parameters from 25 reflections
*b* = 6.7553 (1) Å	θ = 1.5–27.0º
*c* = 9.6222 (1) Å	µ = 3.66 mm^−^^1^
α = 104.325 (1)º	*T* = 293 (2) K
β = 100.604 (1)º	Parallelepiped, brown
γ = 101.696 (1)º	0.4 × 0.07 × 0.03 mm
*V* = 402.625 (9) Å^3^	

### Data collection

**Table:**

Enraf–Nonius CAD-4 diffractometer	*R*_int_ = 0.049
Radiation source: fine-focus sealed tube	θ_max_ = 27.0º
Monochromator: graphite	θ_min_ = 2.3º
*T* = 293(2) K	*h* = −8→8
ω/2θ scans	*k* = −8→8
Absorption correction: ψ scan(North *et al.*, 1968)	*l* = −12→12
*T*_min_ = 0.871, *T*_max_ = 0.965	2 standard reflections
3118 measured reflections	every 120 min
1712 independent reflections	intensity decay: 0.4%
1415 reflections with *I* > 2σ(*I*)	

### Refinement

**Table:**

Refinement on *F*^2^	Secondary atom site location: difference Fourier map
Least-squares matrix: full	*w* = 1/[σ^2^(*F*_o_^2^) + (0.0426*P*)^2^ + 0.4338*P*] where *P* = (*F*_o_^2^ + 2*F*_c_^2^)/3
*R*[*F*^2^ > 2σ(*F*^2^)] = 0.033	(Δ/σ)_max_ < 0.001
*wR*(*F*^2^) = 0.095	Δρ_max_ = 0.73 e Å^−^^3^
*S* = 1.06	Δρ_min_ = −1.18 e Å^−^^3^
1712 reflections	Extinction correction: SHELXL97 (Sheldrick, 2008), Fc^*^=kFc[1+0.001xFc^2^λ^3^/sin(2θ)]^-1/4^
146 parameters	Extinction coefficient: 0.003 (2)
Primary atom site location: structure-invariant direct methods	

### Special details

**Table:**

Geometry. All e.s.d.'s (except the e.s.d. in the dihedral angle between two l.s. planes) are estimated using the full covariance matrix. The cell e.s.d.'s are taken into account individually in the estimation of e.s.d.'s in distances, angles and torsion angles; correlations between e.s.d.'s in cell parameters are only used when they are defined by crystal symmetry. An approximate (isotropic) treatment of cell e.s.d.'s is used for estimating e.s.d.'s involving l.s. planes.
Refinement. Refinement of *F*^2^ against ALL reflections. The weighted *R*-factor *wR* and goodness of fit *S* are based on *F*^2^, conventional *R*-factors *R* are based on *F*, with *F* set to zero for negative *F*^2^. The threshold expression of *F*^2^ > σ(*F*^2^) is used only for calculating *R*-factors(gt) *etc*. and is not relevant to the choice of reflections for refinement. *R*-factors based on *F*^2^ are statistically about twice as large as those based on *F*, and *R*- factors based on ALL data will be even larger.

### Fractional atomic coordinates and isotropic or equivalent isotropic displacement parameters (Å^2^)

**Table:**

	*x*	*y*	*z*	*U*_iso_*/*U*_eq_	
V1	0.11192 (10)	0.04819 (10)	0.30906 (7)	0.00700 (18)	
V2	0.23427 (10)	−0.33675 (10)	−0.04757 (7)	0.00795 (18)	
V3	−0.22756 (10)	−0.42610 (10)	0.38667 (7)	0.00858 (19)	
Mg1	−0.20718 (18)	−0.18407 (18)	−0.01815 (13)	0.0043 (3)	
Mg2	−0.76019 (19)	−0.4331 (2)	0.29306 (14)	0.0098 (3)	
Na	0.6408 (4)	0.0239 (3)	0.3640 (3)	0.0416 (6)	
O1	0.0677 (4)	0.1930 (5)	0.4803 (3)	0.0156 (6)	
O2	0.2277 (4)	0.2383 (4)	0.2402 (3)	0.0118 (6)	
O3	−0.2797 (5)	−0.6006 (5)	0.4778 (3)	0.0172 (6)	
O4	−0.1271 (4)	−0.0914 (5)	0.2063 (3)	0.0143 (6)	
O5	0.0972 (4)	−0.4778 (4)	−0.2430 (3)	0.0118 (6)	
O6	0.2690 (5)	−0.1090 (5)	0.3424 (3)	0.0163 (6)	
O7	0.2151 (4)	−0.5009 (4)	0.0627 (3)	0.0130 (6)	
O8	−0.4437 (4)	−0.3693 (5)	0.3187 (3)	0.0159 (6)	
O9	0.4808 (5)	−0.2374 (5)	−0.0409 (3)	0.0188 (7)	
O10	0.1292 (4)	−0.1322 (4)	0.0086 (3)	0.0116 (5)	

### Atomic displacement parameters (Å^2^)

**Table:**

	*U*^11^	*U*^22^	*U*^33^	*U*^12^	*U*^13^	*U*^23^
V1	0.0082 (3)	0.0063 (3)	0.0071 (3)	0.0016 (2)	0.0016 (2)	0.0034 (2)
V2	0.0112 (3)	0.0072 (3)	0.0066 (3)	0.0036 (2)	0.0021 (2)	0.0032 (2)
V3	0.0110 (3)	0.0083 (3)	0.0078 (3)	0.0031 (2)	0.0034 (2)	0.0033 (2)
Mg1	0.0063 (6)	0.0029 (5)	0.0037 (6)	0.0009 (4)	0.0012 (4)	0.0014 (4)
Mg2	0.0104 (7)	0.0096 (6)	0.0093 (6)	0.0022 (5)	0.0016 (5)	0.0033 (5)
Na	0.0371 (13)	0.0245 (11)	0.0622 (16)	0.0043 (9)	0.0221 (11)	0.0066 (11)
O1	0.0171 (15)	0.0156 (14)	0.0122 (14)	0.0003 (11)	0.0059 (11)	0.0020 (11)
O2	0.0143 (14)	0.0099 (13)	0.0114 (13)	0.0023 (10)	0.0029 (11)	0.0045 (11)
O3	0.0259 (16)	0.0128 (14)	0.0188 (15)	0.0092 (12)	0.0101 (13)	0.0087 (12)
O4	0.0123 (14)	0.0150 (14)	0.0136 (14)	−0.0011 (11)	0.0024 (11)	0.0053 (11)
O5	0.0140 (14)	0.0133 (14)	0.0101 (13)	0.0054 (11)	0.0050 (11)	0.0043 (11)
O6	0.0184 (15)	0.0149 (14)	0.0198 (15)	0.0075 (11)	0.0059 (12)	0.0092 (12)
O7	0.0150 (14)	0.0129 (14)	0.0124 (13)	0.0059 (11)	0.0014 (11)	0.0057 (11)
O8	0.0124 (14)	0.0175 (14)	0.0190 (15)	0.0051 (11)	0.0034 (11)	0.0069 (12)
O9	0.0173 (16)	0.0167 (15)	0.0241 (17)	0.0046 (12)	0.0073 (13)	0.0074 (13)
O10	0.0127 (13)	0.0097 (13)	0.0142 (13)	0.0038 (10)	0.0041 (11)	0.0054 (11)

### Geometric parameters (Å, °)

**Table:**

V1—O4	1.672 (3)	Mg2—Mg1^i^	3.1470 (17)
V1—O6	1.688 (3)	Mg2—V2^i^	3.4044 (14)
V1—O2	1.704 (3)	Mg2—Na^iv^	3.492 (3)
V1—O1	1.806 (3)	Mg2—Na^ii^	3.575 (3)
V2—O9	1.643 (3)	Na—O3^viii^	2.404 (4)
V2—O10	1.694 (3)	Na—O6	2.435 (4)
V2—O7	1.717 (3)	Na—O4^ix^	2.477 (4)
V2—O5	1.848 (3)	Na—O8^ix^	2.509 (4)
V2—Mg1	3.3760 (14)	Na—O6^x^	2.665 (4)
V2—Mg2^i^	3.4044 (14)	Na—O1^ix^	2.771 (4)
V3—O8	1.642 (3)	Na—V1^ix^	3.291 (2)
V3—O3	1.655 (3)	Na—V3^ix^	3.380 (2)
V3—O1^ii^	1.757 (3)	Na—V1^x^	3.474 (3)
V3—O5^iii^	1.827 (3)	Na—Mg2^ix^	3.492 (3)
V3—Na^iv^	3.380 (2)	Na—Na^x^	3.543 (5)
V3—Na^v^	3.582 (2)	O1—V3^ii^	1.757 (3)
Mg1—O9^iv^	2.020 (3)	O1—Na^iv^	2.771 (4)
Mg1—O4	2.028 (3)	O2—Mg1^vi^	2.049 (3)
Mg1—O2^vi^	2.049 (3)	O2—Mg2^viii^	2.132 (3)
Mg1—O7^iii^	2.051 (3)	O3—Mg2^vii^	2.120 (3)
Mg1—O10^vi^	2.069 (3)	O3—Na^v^	2.404 (4)
Mg1—O10	2.178 (3)	O4—Na^iv^	2.477 (4)
Mg1—Mg2^i^	3.1470 (17)	O5—V3^iii^	1.827 (3)
Mg1—Mg1^vi^	3.240 (2)	O5—Mg2^i^	2.156 (3)
Mg1—V1^vi^	3.2839 (13)	O6—Mg2^ix^	2.082 (3)
Mg2—O8	2.043 (3)	O6—Na^x^	2.665 (4)
Mg2—O6^iv^	2.082 (3)	O7—Mg1^iii^	2.051 (3)
Mg2—O7^iv^	2.117 (3)	O7—Mg2^ix^	2.117 (3)
Mg2—O3^vii^	2.120 (3)	O8—Na^iv^	2.509 (4)
Mg2—O2^v^	2.132 (3)	O9—Mg1^ix^	2.020 (3)
Mg2—O5^i^	2.156 (3)	O10—Mg1^vi^	2.069 (3)
			
O4—V1—O6	112.10 (15)	O10^vi^—Mg1—O10	80.59 (12)
O4—V1—O2	113.54 (14)	O8—Mg2—O6^iv^	88.16 (13)
O6—V1—O2	111.46 (14)	O8—Mg2—O7^iv^	86.61 (12)
O4—V1—O1	104.42 (14)	O6^iv^—Mg2—O7^iv^	98.45 (12)
O6—V1—O1	110.07 (14)	O8—Mg2—O3^vii^	90.45 (13)
O2—V1—O1	104.72 (14)	O6^iv^—Mg2—O3^vii^	88.10 (13)
O9—V2—O10	107.80 (14)	O7^iv^—Mg2—O3^vii^	172.73 (13)
O9—V2—O7	110.35 (15)	O8—Mg2—O2^v^	88.64 (13)
O10—V2—O7	111.25 (14)	O6^iv^—Mg2—O2^v^	176.66 (13)
O9—V2—O5	107.91 (14)	O7^iv^—Mg2—O2^v^	80.40 (12)
O10—V2—O5	107.49 (13)	O3^vii^—Mg2—O2^v^	92.89 (12)
O7—V2—O5	111.87 (13)	O8—Mg2—O5^i^	173.94 (13)
O8—V3—O3	109.72 (15)	O6^iv^—Mg2—O5^i^	95.28 (12)
O8—V3—O1^ii^	106.39 (15)	O7^iv^—Mg2—O5^i^	87.95 (11)
O3—V3—O1^ii^	106.04 (14)	O3^vii^—Mg2—O5^i^	94.64 (12)
O8—V3—O5^iii^	111.97 (14)	O2^v^—Mg2—O5^i^	87.83 (11)
O3—V3—O5^iii^	111.02 (14)	O3^viii^—Na—O6	105.48 (13)
O1^ii^—V3—O5^iii^	111.43 (13)	O3^viii^—Na—O4^ix^	115.05 (13)
O9^iv^—Mg1—O4	96.22 (13)	O6—Na—O4^ix^	131.92 (14)
O9^iv^—Mg1—O2^vi^	94.40 (13)	O3^viii^—Na—O8^ix^	163.72 (16)
O4—Mg1—O2^vi^	168.05 (13)	O6—Na—O8^ix^	70.96 (12)
O9^iv^—Mg1—O7^iii^	93.58 (13)	O4^ix^—Na—O8^ix^	76.24 (12)
O4—Mg1—O7^iii^	100.80 (12)	O3^viii^—Na—O6^x^	70.20 (12)
O2^vi^—Mg1—O7^iii^	83.95 (12)	O6—Na—O6^x^	92.11 (12)
O9^iv^—Mg1—O10^vi^	100.55 (13)	O4^ix^—Na—O6^x^	124.66 (14)
O4—Mg1—O10^vi^	88.02 (12)	O8^ix^—Na—O6^x^	93.84 (13)
O2^vi^—Mg1—O10^vi^	84.66 (12)	O3^viii^—Na—O1^ix^	69.27 (11)
O7^iii^—Mg1—O10^vi^	162.47 (13)	O6—Na—O1^ix^	162.23 (15)
O9^iv^—Mg1—O10	178.74 (12)	O4^ix^—Na—O1^ix^	62.91 (10)
O4—Mg1—O10	83.25 (12)	O8^ix^—Na—O1^ix^	109.07 (13)
O2^vi^—Mg1—O10	86.24 (12)	O6^x^—Na—O1^ix^	70.12 (11)
O7^iii^—Mg1—O10	85.40 (11)		

Symmetry codes: (i) −*x*−1, −*y*−1, −*z*; (ii) −*x*, −*y*, −*z*+1; (iii) −*x*, −*y*−1, −*z*; (iv) *x*−1, *y*, *z*; (v) *x*−1, *y*−1, *z*; (vi) −*x*, −*y*, −*z*; (vii) −*x*−1, −*y*−1, −*z*+1; (viii) *x*+1, *y*+1, *z*; (ix) *x*+1, *y*, *z*; (x) −*x*+1, −*y*, −*z*+1.
